# Parkinson's Disease: Leucine-Rich Repeat Kinase 2 and Autophagy, Intimate Enemies

**DOI:** 10.1155/2012/151039

**Published:** 2012-08-30

**Authors:** José M. Bravo-San Pedro, Rubén Gómez-Sánchez, Elisa Pizarro-Estrella, Mireia Niso-Santano, Rosa A. González-Polo, José M. Fuentes Rodríguez

**Affiliations:** Centro de Investigación Biomédica en Red sobre Enfermedades Neurodegenerativas (CIBERNED), Departamento de Bioquímica y Biología Molecular y Genética, E. Enfermería y T.O., Universidad de Extremadura, 10003 Cáceres, Spain

## Abstract

Parkinson's disease is the second common neurodegenerative disorder, after Alzheimer's disease. It is a clinical syndrome characterized by loss of dopamine-generating cells in the substancia nigra, a region of the midbrain. The etiology of Parkinson's disease has long been through to involve both genetic and environmental factors. Mutations in the leucine-rich repeat kinase 2 gene cause late-onset Parkinson's disease with a clinical appearance indistinguishable from Parkinson's disease idiopathic. Autophagy is an intracellular catabolic mechanism whereby a cell recycles or degrades damage proteins and cytoplasmic organelles. This degradative process has been associated with cellular dysfunction in neurodegenerative processes including Parkinson's disease. We discuss the role of leucine-rich repeat kinase 2 in autophagy, and how the deregulations of this degradative mechanism in cells can be implicated in the Parkinson's disease etiology.

## 1. Parkinson's Disease

The ability to control body movement is an inherent human capacity. It is difficult to imagine the normal performance of many daily and routine activities without a normal control of movement. Nevertheless, many people experience body movement disorders and struggle daily with their handicap. Since antiquity, there have been a multitude of references to individuals with movement disorders. Galen and Hippocrates described people who presented classic symptoms of Parkinson's in ancient Greece. References to the disease also occur in the papyrus writings of the Egyptians of the 19th dynasty and the classic Chinese texts of the 1st century BC. 

However, it was not until 1817 that James Parkinson (1755–1824), a British physician with ample clinical experience, published *“An Essay on the Shaking Palsy*.*”* PD is the second common neurodegenerative disorder, after Alzheimer's disease. Estimated prevalence rate is about 300/100,000 population and incidence and prevalence rates rise with advancing age [1]. Initial symptoms, which typically begin at or around age 60, reaching an important disability within 5 or 15 years later [2]. The origin of the disorder lies in the loss of at least 50% of the neurons in an area of the mesencephalon known as the substantia nigra pars compact. These neurons show a characteristic dark pigmentation because of the presence of melanin. Under normal physiological conditions, these neurons produce dopamine, which provides inhibitory signals to the corpus striatum to control the execution of smooth and precise movements. In a person with Parkinson's, the death of neurons in the substantia nigra leads to a depletion of dopamine in the corpus striatum [3], which is responsible for the patients' motor symptoms, especially akinesia [4].

Over time, PD has been suggested to have a multifactorial etiology, in which both genetic and environmental factors are included [5]. In 1988, Gowers introduced the possibility of a hereditary basis for PD, given the family history of a considerable number of patients with the disease. Therefore, knowledge about the genetic factors involved in the disease is essential when clarifying the possible causes and mechanisms underlying its development. Epidemiological studies have revealed that most cases of individuals with the illness are sporadic and that only 5–10% shows a pattern of hereditary transmission, which highlights the importance of environmental factors in the origin of the illness. As a result, it is postulated that the cause of the disease can be attributed to an interaction between hereditary and environmental factors, where the genetic factor predisposes but does not determine the development of the illness. A family history of PD constitutes a risk factor at the time of PD development [6]. Family cases of Parkinsonism were observed, which led to an increase in studies evaluating a possible genetic predisposition to developing PD. In 1997, an autosomal dominant mutation of the *PARK1* gene that coded for the *α*-synuclein protein was identified in Italian and Greek families who suffered from a hereditary form of PD [7]. This finding, along with the discovery of *α*-synuclein as the major component of Lewy bodies [8], led to greater interest in the genetic aspects of PD. In the following years, other genes implicated in PD were discovered (Table 1). In 1998, the *PARK2* gene, which codes for the parkin protein [9], was identified; it was found to be mutated in an inherited juvenile variation of PD. Subsequent studies identified new key mutations in PD, such as the mutation of the DJ-1 protein in Dutch and Italian families [10], which is responsible for an autosomal recessive variation of PD. A mutation in the *PARK6* gene coding for the PINK1 protein has been described; the mutation could originate from a metabolic error and neuronal death in the substantia nigra [11]. In recent years, the number of studies related to the *PARK8* gene, which codes for the leucine-rich repeat kinase 2 (LRRK2) protein and could be directly associated with the development of PD, has risen dramatically.

## 2. Leucine-Rich Repeat Kinase 2

In 2004, mutations in the *PARK8* gene were described as one of the major genetic causes associated with hereditary Parkinsonism [12]. The *PARK8* gene was studied for the first time in the Japanese Sagamihara family; members who suffered from PD responded positively to treatment with L-Dopa and had idiopathic Parkinsonism disease characteristics [13]. This protein was later associated with PD by studies in two other families (German and Canadian) who also presented late-onset hereditary autosomal dominant Parkinsonism [14].

The *PARK8* gene is located on the 12q12 chromosome and has 51 exons that code for a 2527 amino acid protein with molecular weight of 285 kDa. This protein has multiple denominations, including PARK8, RIPK7, or ROCO2. However, the most utilized names are *leucine-rich repeat kinase* 2 (LRRK2) because of the presence of a domain rich in leucine, or *dardarin* (from the Basque word *dardara*, which means trembling, one of the most characteristic symptoms of PD).

**Figure 1:**
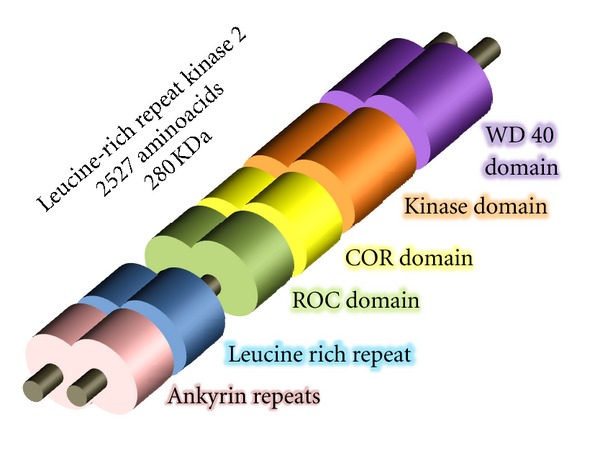
LRRK2 domain structure with homodimeric conformation. LRRK2 is a protein that contains ankyrin repeats, leucine-rich repeats, a catalytic core of the protein contains a GTP-binding ROC (Ras of complex proteins), COR domain (C-terminal of ROC), kinase domain. At the C-terminus is a WD40 repeat followed by a short C-terminal tail.

LRRK2 (Figure 1) is a protein that has a homodimer structure [15], which suggests that it could have the capacity to self-regulate its kinase activity and GTPase activity [16]. Recent studies have indicated that LRRK2 is predominantly found in monomer form and that it only takes a homodimer configuration to regulate enzymatic activity [17]. LRRK2 contains multiple conserved domains including Ankyrin, leucine-rich repeat (LRR), WD40, a MAPKKK kinase, and GTPase.

More than 20 mutations are known in the LRRK2 structure [18] and mutations studied most relevant in the LRRK2 structure, *G2019S,* and *R1441*, are locates the kinase and GTPase domain, respectively. The *G2019S* mutation shows reduced penetrance (as low as 24%), however, *R1441* mutation is highly penetrant (95% at older ages) [19].

Various studies have associated changes in LRRK2 kinase activity with cellular death processes. The kinase domain of LRRK2 is highly homologous with other MAPKKKs of the tyrosine-kinase group [20], in which various mutations have been detected. These mutations have been mostly found in the preserved DF/YG sequence, which has been linked to PD. The G2019S mutation is found in the Mg^2+^ union site of the kinase domain. The exchange of glycine for serine facilitates the access of the kinase domain to its substrates, thereby augmenting its capacity for autophosphorylation 2.5-fold and its capacity to phosphorylate other substrates 3-fold. The I2020T mutation is found in the zone adjacent to the 2019 residue, and it therefore influences the activation site of the kinase domain. The exchange of an isoleucine for a tyrosine next to the DYG activation site increases the autophosphorylation capacity of LRRK2 by 40%. Such a mutation can also modify the specificity for substrates and result in an increase in toxicity [21].

### 2.1. Functions LRRK2

LRRK2 is expressed in organs within the central nervous system and outside the central nervous system, including the kidneys, lungs, liver, heart, and leukocytes [22]. LRRK2 is expressed in the different areas of the brain, with ample expression in the cortex, the basal ganglia, the cerebellum, and the hippocampus [23]. It is also present in the substantia nigra of the mesencephalon, although at low levels [24]. Thus, LRRK2 is found in areas that contain dopaminergic neurons. The interruption of dopamine transmission does not affect the expression of LRRK2, although it is not known how this change affects the functionality of the protein. Curiously, an increase in the expression of LRRK2's mRNA has been observed upon stimulation of MPTP [25]. LRRK2 is primarily a cytosolic protein, although 10% of the protein is located in the external membrane of the mitochondria [23]. LRRK2 is also associated with the plasma membrane, the Golgi apparatus, microtubules [26], synaptic vesicles [27], and lipid rafts [28].

Because of the number of domains in its structure, the LRRK2 protein can interact with various other proteins. According to Dächsel et al., 3 groups of proteins can interact with LRRK2: the chaperone-mediated response group, the cytoskeletal interaction group, and the kinase activity proteins [29]. However, previous studies discovered multiple new proteins that also interact with LRRK2, including *β*-tubulin and actin, which interact with the Roc domain of LRRK2 independently of GTP, and are considered kinase substrates of LRRK2 [30]. As such, LRRK2 could be implicated in the reorganization processes of the cytoskeleton [31].

When we inhibit the interaction between LRRK2 and Hsp90 (heat shock protein 90), which is responsible for the regulation of the folding of other proteins, the degradation of Hsp90 is mediated by proteasomes. Therefore, Hsp90 could be responsible for maintaining the stability of LRRK2. Following an alteration of this stability, the elimination of LRRK2 occurs. In the case of mutations that compromise cellular viability, this destabilization could be utilized to degrade the molecule that is causing the cellular damage, as is the case with the G2019S mutation of LRRK2 [32]. CHIP (Hsp70-interaction protein) is another protein that has been studied for its interaction with LRRK2 [33] and that could affect the molecular stability of LRRK2. Similar interaction exist with the 14.3.3 proteins that are directly implicated in the maintenance of the stability of LRRK2 [34], which is dependent upon the LRRK2's autophosphorylation capacity [35].

LRRK2 can also influence cellular death processes because of its interaction with proteins such as FADD (Fas-associated protein with dead domain), which is implicated in the activation of apoptosis. Recent studies have indicated a relationship between LRRK2 and the activation of programmed cellular death, which suggests that FADD/caspase 8 contributes to the cellular death induced by LRRK2 [36].

Rab5b is implicated in the regulation of endocytosis and interacts with LRRK2. It could play a fundamental role in the synaptic function that modulates the endocytosis of synaptic vesicles [27].

Several studies have associated LRRK2 with other proteins related to PD, such as parkin [37], PINK-1, and DJ-1 [38]. Studies have also related LRRK2 to *α*-synuclein, indicating that an increase in LRRK2 produces an acceleration of neuropathologies caused by mutations in *α*-synuclein [39].

The interactions of LRRK2 with MAPKs such as ERK (kinases activated by extracellular signals) [40], JNK (N-terminal of C-Jun kinases), and p38 [41] have also been studied, especially with regard to the transport of proteins through synaptic vesicles [27] and the process of ubiquitination [33]. Some studies have also associated LRRK2 with autophagy [42] and apoptosis [36].

## 3. Autophagy

The maintenance of the correct balance between the synthesis and degradation of all cellular constituents is vital for the survival of the cell. The cell maintains a continual process of renewing its organelles and proteins, and it is necessary to discard the material that has been synthesized but is no longer useful to the cell. The unneeded material is degraded and reused to obtain energy or synthesize new molecules. The cell has two primary mechanisms for breaking down cellular components: the ubiquitin-proteasome system [43] and autophagy [44].

**Figure 2:**
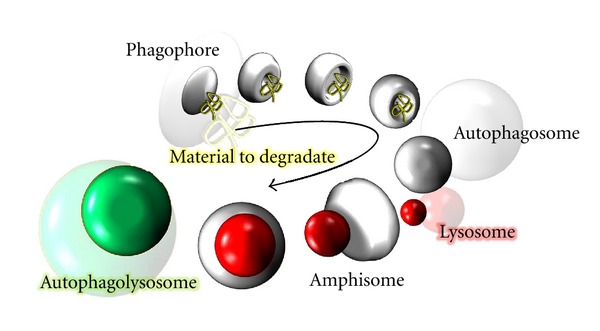
Schematic Illustration on 3D of the autophagy flux. The first step consists of the formation of isolation membranes (phagophore) and elongation of this membrane for sequester the material to degraded (autophagosome). Finally a lysosome is fused with the autophagosome (autophagolysosome) and the cargo is degraded.

The term autophagy is derived from two Greek words: “auto,” which means self, and “phagia,” which indicates the action of eating (autophagy literally means “to eat oneself”). Autophagy is a catabolic intracellular mechanism that has been highly preserved throughout evolution; it is the process by which the cell recycles or degrades proteins or damaged cytoplasmic organelles (Figure 2) [45]. Autophagy was described by Christian de Duve in the 1960s, however, it was not until the 1990s that the genes involved in the process were identified in yeast. Since then, these genes have been termed *Atgs* genes (autophagy-related genes) [46]. Currently, the number of papers published annually on autophagy is exponentially growing because studies are revealing the importance of this mechanism in development and in various illnesses.

An important role of autophagy has been described in neonatal development [47] and in illnesses such as cancer [48], cardiomyopathies [49], musculoskeletal problems, diseases of adipose tissue, and neurodegenerative processes [50, 51]. In fact, it has been described dysfunctional autophagy as one of the failing cellular mechanisms involved in the pathogenesis of idiopathic PD [52]. Studies have also associated autophagy with aging. It has been observed that a hypercaloric diet accelerates the aging process compared with a calorie-restricted diet but not malnourishment. Individuals with a hypocaloric diet had fewer incidences of cancer, cardiovascular disease, and diabetes, and they had a later mortality [53].

**Figure 3:**
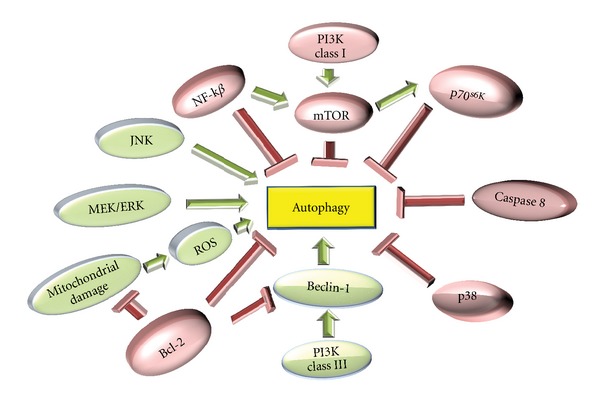
Molecular regulation of autophagy. In the figure, the factors that stimulate autophagy (green) are JNK, ERK1/2, ROS, or PI3K class III, whereas the inhibitory factors (red) are NF-*κ*B, mTOR, caspase 8, Bcl-2, or p38.

Therefore, the importance of the correct regulation of autophagy for maintaining cell viability is clear. However, autophagy involves a complex regulation of cellular recycling (Figure 3). Despite the research efforts undertaken in recent years, many gaps remain in the understanding of the exact regulatory mechanism of autophagy.

The existence of various negative regulators of autophagy is known, among which the mTOR (the mammalian target of rapamycin) protein is one of the most studied autophagy repressors. mTOR is a protein kinase that is active under favorable cellular conditions, repressing autophagy through the phosphatase PP2A [54]. The phosphoinositido3-kinase (PI3K) class I route is also implicated in the negative regulation of autophagy through direct interactions with mTOR [55]. Like PI3k class I, NF-*κ*B exercises negative regulation by activating mTOR [56]. Another molecule that negatively regulates autophagy is Bcl-2.I, which can inhibit the activation route via the PI3K class III pathway (through interactions with Beclin-1) and through the protection provided by Bcl-2 to the mitochondrial membrane of the cell [57].

However, many pathways are capable of positively regulating autophagy. The most well-known pathway is the PI3K class III Beclin-1-dependent route, which has been implicated in the activation of the first formation phases of autophagosomes [58]. The stimulation of autophagy by ERK pathway is known [59], and in recent studies, the presence of reactive oxygen species (ROS) has been involved in the regulation of autophagy [60].

## 4. Autophagy-LRRK2

The role of LRRK2 in such complex regulation is complicated. However, certain information is available that directly implicates it in the regulation of this cellular degradation mechanism. The first indication of this possible interaction was the discovery that an endogenous part of LRRK2 is anchored to membranous structures of the cell, including the ER and endosomes [23], and that the overexpression of the mutant form of *G2019S* of LRRK2 in neuronal cells induces the accumulation of autophagic structures [42], as also observed in nonneuronal cells [61], iPSC-based model [62] or transgenic mice [63]. However, LRRK2 interacts with various proteins that are implicated in the regulation of autophagy, such as CAMKK-*β*/AMPK, which is dependent on Ca^2+^ and can induce the accumulation of autophagosomes [64]. In *in vivo* studies, a depletion of LRRK2 is related to a decrease in 4EBP, which is the target of mTOR [65]. This finding directly associates the LRRK2 protein with aging and autophagy processes. However, interestingly, has been observed a age-dependent bi-phasic alteration in autophagic activity in LRRK2 knockout accompanied by modulations in levels of lysosomal proteins and proteases at different months of age [66].

From the studies previously indicated, it is obvious that the LRRK2 protein participates in the regulation of the autophagic cellular process, and as changes in protein activity affect the deregulation of autophagy, it becomes harmful for the cell. Nevertheless, the exact mechanism of the regulation is still unknown.

There are different pathways in the regulation of autophagy in which the LRRK2 protein is involved.

### 4.1. Regulation of Autophagy by Nutrient Deprivation

An equilibrium between the energy available for the cell and the supply of nutrients is essential for cellular survival. In conditions of cellular nutrient deprivation, an increase in the levels of autophagy dependent on the inhibitory protein mTOR is induced to obtain energy by recycling the cell's own components. Many proteins participate in maintaining this equilibrium. The AMPK/mTOR/ULK1 route is one of the most widely studied pathways in terms of the cellular response to energy changes [67]. In the case of energy deficiency, the AMPK protein is responsible for inhibiting the TORC1 complex and activating the autophagy-initiating complex ULK1/Atg13/FIP200. Thus, AMPK participates directly in the regulation of autophagy by nutrient deprivation. It has been confirmed that LRRK2 and AMPK have a close relationship and a Ca^2+^-dependent ability to induce the accumulation of autophagosomes [64]. In addition, LRRK2 siRNA induces an increase in autophagic activity and prevents the cellular death that is caused when autophagy is inhibited, which occurs in states of energy deficiency [61]. Moreover, ULK1/2 is a protein that participates in the regulation of the initial phases of autophagy and has been identified to play a role in the interaction with LRRK2, which could be responsible for the increase in autophagy when an increase in LRRK2 kinase activity is present [17]. Therefore, it appears that the LRRK2 protein can truly intervene in the regulation of the initial phases of autophagy and the induction of autophagy via nutrient deprivation.

### 4.2. Regulation of Nonclassic Autophagy Independent of Beclin-1

 Alternative mechanisms of autophagy induction have been studied in which the classic autophagy protein Beclin-1 does not actively participate. The autophagy observed after treatments with MPTP corresponds to this pattern of autophagy independent of Beclin-1, as it has been observed that the autophagy does not revert after the use of Beclin-1 siRNA [68]. Furthermore, it has been demonstrated that MPTP provokes an increase in the expression of LRRK2 in neurons in the striatum [25], which could be related to an increase in autophagic activity of the cells after treatment with MPTP. However, there are contradictory results, as some studies have shown that the inhibition of this nonclassic autophagy independent of Beclin-1 protects the cell [39]. Others have indicated that the toxicity did not depend on or exacerbate the autophagy arising from increased LRRK2 expression, as there was no significant difference in the sensitivity to MPTP between wild type and LRRK2 knockout mice [69]. Therefore, further studies are needed to elucidate the relationship between the increase in LRRK2 protein expression and Beclin-1 independent autophagy and to identify how this relationship can influence the sensitivity of the cells.

### 4.3. Regulation of the Stability of the Cytoskeleton by LRRK2 and Its Importance in Autophagy

Studies focusing on the control of the quality of material that is degraded by autophagy have revealed the importance of proteins such as HDAC6 and actin for the maturation and completion of autophagy [70]. Many studies focused on the role of LRRK2 in the reorganization and functional stability of the cytoskeleton. LRRK2 phosphorylates proteins directly, such as heterotetramers of *α*/*β*-tubulin [30] and actin [31] or indirectly, such as moesin [71], ezrin, and radixin [72]. These proteins are essential for the regulation of actin activity, which suggests that LRRK2 is a regulator of cytoskeletal stability and an essential factor for efficient autophagy. One recent study indicated that the overexpression of Rac1 attenuated the disassembly of the actin filaments in cells with G2019S mutations of LRRK2 [73], which supports the importance of LRRK2 activity in the correct assembly of the cytoskeleton.

### 4.4. Regulation of Autophagy Mediated by the MAPK p42/44 Pathway

MAPKs, JNK, and ERK1/2 are associated with positive regulatory processes of autophagy [40, 59, 74]. Recently, MAPKs have been documented as LRRK2 substrates [75]. In fact, an increase in the levels of ERK1/2 activity has been observed in cells that overexpress LRRK2 or its mutant forms G2019S and R1441C [35]. Studies that utilized pharmacological MEK/ERK1/2 route inhibitors such as U0126 revealed that the inhibition of this pathway hinders neurite retraction and exacerbates autophagy in cells with the G2019S LRRK2 mutation [40, 42]. Moreover, the sensitivity of cells is increased by the G2019S mutation when an increase in oxidative stress is present; this greater toxicity can be reverted through the use of the pharmacological MEK/ERK1/2 route inhibitor U0126 [34]. For this reason, the exacerbated autophagy that is produced by increased kinase activity of LRRK2, in which the MAPK ERK1/2 pathway actively participates, can be detrimental to the cell by increasing its sensitivity to oxidative stress [40]. In this sense many studies show that *G2019S* LRRK2 mutation induces alpha-synuclein aggregation, initiating and enhancing the formation of alpha-synuclein aggregates [76]. Moreover, this interaction is MEK/ERK pathway dependent [35], although this mechanism still remains unknown [74, 77–79]. Therefore, the defensive or protective autoregulatory mechanism that accelerates the degradation of misfolded proteins may explain the increased number of autophagic vacuoles in the brains of PD patients [80] and is possible than these exacerbated levels to be a critical contributing factor in the induction of cell death [81].

## 5. Conclusions and Future Perspectives

**Figure 4:**
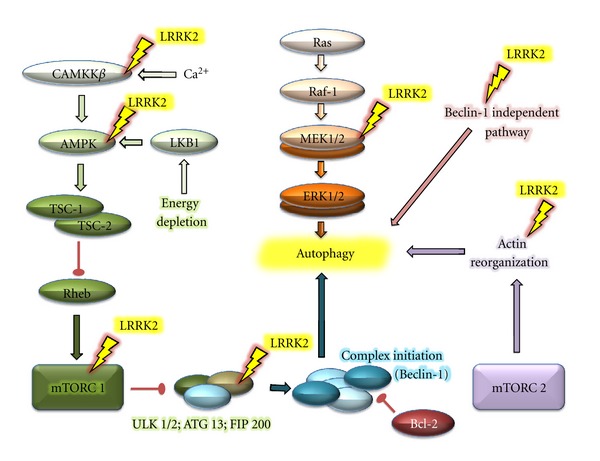
Diagram with the possible sites of interaction LRRK2-autophagy. The figure shows the different routes involved in the regulation of autophagy that may be LRRK2 dependent.

There is evidence of deregulated autophagy processes in neurons of the substantia nigra in PD patients. Thus, it is logical that deregulation could intervene, at least in part, in the etiology of PD [82]. The deregulation of autophagy has been associated with the LRRK2 protein. Deregulation is usually associated with the modulation of the activities of the protein, especially kinase activity. Some studies also indicate that the inhibition of LRRK2 kinase activity can protect against neuronal toxicity created by the *G2019S* mutation of LRRK2 [83], which is also responsible for the increase in autophagy levels. Furthermore, studies have indicated that LRRK2 is essential for the development of effective autophagy (Figure 4), as it is directly related to the cytoskeleton and cell membranes. Therefore, alterations in the kinase activity could deregulate this cell degradation mechanism and become toxic to the cell. Finally, LRRK2 could be involved in cell autophagy in response to stimuli such as deprivation, the generation of ROS, or drugs such as MPTP by making cells with LRRK2 dysfunction more sensitive to these stimuli.

LRRK2 protein is involved in cellular autophagy through direct modulation, the alteration of its own kinase activity, or the mediation of autophagy in response to external stimuli. The LRRK2 protein is also essential for maintaining the equilibrium between cellular degradation and synthesis.

Therefore, it is important to understand the activity of LRRK2 to elucidate the cellular death that has been identified in studies of PARK8 mutations. This knowledge is essential for the development of strategies for reducing the cellular sensitivity and cell death that could trigger the development of PD.

## Figures and Tables

**Table 1:** Genes associated with Parkinson's disease linkage.

Gene	Locus	Protein name	Inheritance pattern	Description
*PARK 1/4*	4q21.3-q22	*α*-synuclein (SNCA)	AD	Lewy's body component
*PARK 2*	6q25.2-27	Parkin	AR	E3 ubiquitin-protein ligase
*PARK 3*	2p13	*¿*?	AD	*¿*?
*PARK 5*	4p14	UCH-L1	AD	Ubiquitin C-terminal hydrolase
*PARK 6*	1p35-36	PINK1	AR	Mitochondrial kinase
*PARK 7*	1p36	DJ-1	AR	Chaperone mitochondrial kinase
*PARK 8*	12q12	LRRK2	AD	Kinase/GTPase
*PARK 9*	1p36	ATP13A2	AR	Cationic transport
*PARK 10*	1p32	*¿*?	AD	*¿*?
*PARK 11*	2q36-q37	GIGYF2	AD	Receptor tyrosine phosphorylation regulation
*PARK 12*	Xq21-q25	*¿*?	X-linked	*¿*?
*PARK 13*	2p13	HTRA2/OMI	AD	Serine protease
*PARK 14*	22q13.1	PLA2G6	AR	Phospholipase A2
*PARK 15*	22q11.2	FBXO7	AR	E3 ubiquitin-protein
*PARK 16*	1q32	*¿*?	*¿*?	ligase*¿*?

AD: autosomal dominant; AR: autosomal recessive.
